# Iodine Status of 6–12-Year-Old Children in Russia over the Past 10 Years: A Scoping Review

**DOI:** 10.3390/nu14040897

**Published:** 2022-02-21

**Authors:** Rimma Korobitsyna, Andrey Aksenov, Tatiana Sorokina, Anna Trofimova, Andrej M. Grjibovski

**Affiliations:** 1 Arctic Biomonitoring Laboratory, Northern (Arctic) Federal University named after M. V. Lomonosov, Naberezhnaya Severnoy Dvini 17, 163002 Arkhangelsk, Russia; a.s.aksenov@narfu.ru (A.A.); t.sorokina@narfu.ru (T.S.); a.trofimova@narfu.ru (A.T.); andrej.grjibovski@gmail.com (A.M.G.); 2 Central Scientific Research Laboratory, Northern State Medical University, Troitskiy Ave. 51, 163000 Arkhangelsk, Russia; 3 West Kazakhstan Marat Ospanov Medical University, Aktobe 030019, Kazakhstan; 4 Department of Epidemiology and Modern Vaccination Technologies, Sechenov First Moscow State Medical University (Sechenov University), Moscow 119991, Russia

**Keywords:** iodine status, iodine deficiency, children, urinary iodine concentration, Russia

## Abstract

Iodine is an essential element for growth and development of children. Ensuring adequate iodine intake and monitoring iodine intake are important public health concerns. According to the World Health Organization, a population-based assessment of iodine status is often done by measuring urine iodine concentration (UIC) in children aged 6–12 years. National data for large countries may hide regional differences in the UIC. Currently, there is limited data on the iodine status of children in Russia. We summarized the evidence on the iodine status of children in Russia using both international and local literature in accordance with the PRISMA guidelines. A total of 2164 studies were identified, 12 of which met the selection criteria and covered 10 of 85 federal subjects. For most of the Russian regions there was no information on UIC. A range of methodologies were used to determine UIC. The median UIC ranged from 46 μg/L in the mountainous areas in the Republic of Kabardino-Balkaria, which corresponds to a moderate iodine deficiency (ID), to 719 μg/L in the town of Turinsk (Sverdlovsk region) indicating excessive of iodine intake. Nationwide monitoring should be implemented in Russia and public health measures should be adjusted to regional and local conditions to ensure adequate iodine nutrition for all citizens.

## 1. Introduction

Iodine is an important nutrient for human growth, particularly during the early stages of development. This essential element plays a crucial role in foetal neurodevelopment, especially deficits leading to poorer cognitive outcomes [1]. Children can be impacted both at different times, both during foetal development in case of iodine deficiency (ID) in the mother, and in childhood with insufficient iodine nutrition (IN) [2]. Even mild ID during pregnancy and childhood can have negative effects [3,4].

An important factor in obtaining information on the IN of children is monitoring in all regions [5]. The Iodine Global Network (IGN) is a resource that reflects the average UIC values in different countries [6]. The national average UIC values may be adequate for small and relatively homogenous countries, but in large countries consisting of regions and territories at different levels of economic development and populated by different ethnic groups, national data may hide large regional variations in UIC. 

According to the IGN, Russia is a country with mild iodine deficiency with the median urinary iodine concentration (mUIC) below 100 μg/L for school-age children [6]. Russia is the largest country in the world and represents a collection of territories with different levels of economic and social development. Due to its size, Russia remains one of the few countries in the world that has not conducted a nationwide study of the prevalence of iodine deficiency. Instead, individual projects were conducted in many regions of the country, including studies on the median iodine concentration in schoolchildren [7]. Most of the results of these studies were published in Russian in local journals limiting international access to the evidence. Moreover, methods of measuring mUIC vary across individual studies threatening the validity of the of available data for Russia. Although prevention of iodine deficiency is regulated in many regions [8], the information on its effectiveness is scarce.

The aim of this study was to summarize the current evidence on the iodine status of 6–12-year-old children in the Russian Federation published in English and Russian peer-reviewed literature that can be used for international comparisons and development of national- and regional evidence-based public health measures.

## 2. Materials and Methods

PRISMA Extension for Scoping Reviews guidelines [9] were applied for selection procedures and presentation of the results. 

### 2.1. Search Strategy

A literature search was performed in PubMed, Scopus and Web of Science, Google Scholar databases as well as in the two largest sources of scientific evidence published in Russian, namely, the National Electronic Library (eLIBRARY.RU) and CyberLeninka. Search terms and combinations of terms in databases included (iodine or iodine deficiency OR iodine status OR iodine content) AND (child OR children OR elementary school children OR primary school children) AND (urine OR median urinary iodine concentration) AND (Russia). Duplicate publications were identified and eliminated.

To provide up-to-date information on the iodine status of children in Russia, the search was limited to studies published from 2013 through 2021.

### 2.2. Record Screening

Duplicates were removed manually. The remaining titles and abstracts were entered into Microsoft Excel as a table. All members of the team had access to the table and participated in the selection process. Full text papers were evaluated by two authors (R.K., A.A.). The senior author (A.M.G) was contacted in all cases of disagreement. 

### 2.3. Selection

The publication was considered eligible if it contained information on:

(i) the study contained data on UIC in children;
(ii) the age of children was between 6 and 12 years;
(iii) the children lived on the territory of the Russian Federation.

Studies on adults, animals, infants (<1 year of age), patients with diagnosed thyroid disease and/or other chronic diseases or patients treated with radioactive iodine isotopes viz. ^123^I, ^124^I, ^125^I, and ^131^I were excluded.

### 2.4. Data Extraction

Information on study design, sample, and main results were extracted by one author (R.K.) into an Excel spreadsheet. The second author (A.A.) checked the data extracted from each publication.

## 3. Results

### 3.1. Publication Selection

**Figure 1:**
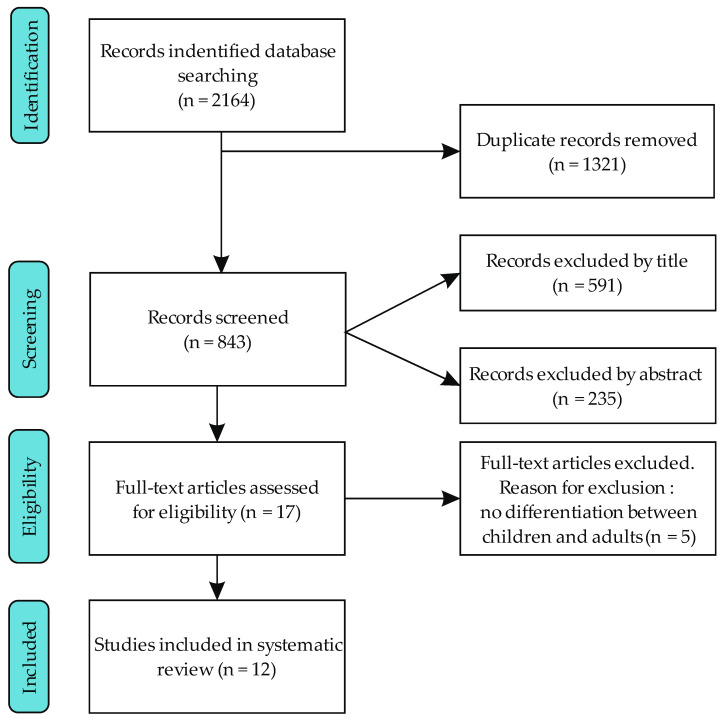
Selection of articles.

Altogether, 2164 publications were identified: PubMed, n = 11; Scopus, n = 7; Web of Science, n = 8; eLIBRARY.RU, n = 937; CyberLeninka, n = 179 and Google Scholar, n = 1022. After removing duplicates (n = 1321), 843 articles were evaluated by title, resulting in eliminating of 591 of them. Abstracts of the remaining articles were analyzed, and 235 papers were excluded. The full text of 17 articles were assessed by two authors (R.K and A.A), with conflicts (n = 2) resolved by consensus and consultations with the last author (A.M.G). After this assessment, 12 articles were selected for the final qualitative synthesis (Figure 1). 

Twelve studies reported the results of empirical studies conducted between 2013 and 2021. All studies were carried out on the territory of the Russian Federation and covered 10 of 85 federal subjects (6 republics and 4 regions). Republic of Bashkortostan was the territory with the most studies over the last 10 years with studies conducted in 2018 [10], 2015 [11] and 2013 [12]. In other regions over the past ten years, there were either one or no studies. Sample size ranged from 97 [13] to 1782 [14]. The age of study participants in all records varied between 6 and 12 years. In a study by Zahohov et al. [14], only girls comprised the sample. Skorodok et al. [15] presented the data on mUIC with stratification by gender while in all other studies no gender-specific data on UIC were available. More information on the studies included in this review is presented in Table 1.

### 3.2. Description of Included Studies

Participants: Most of the studies included children from primary schools. In a half of the studies only children aged 8–10 years were included, although in another half a wider range of ages was used. 

Study Designs: In most studies the study designs were poorly defined in terms of selection of settings, schools and individual participants, response rates, justification of sample size thus threatening the external validity of the studies [10,11,12]. 

**Figure 2:**
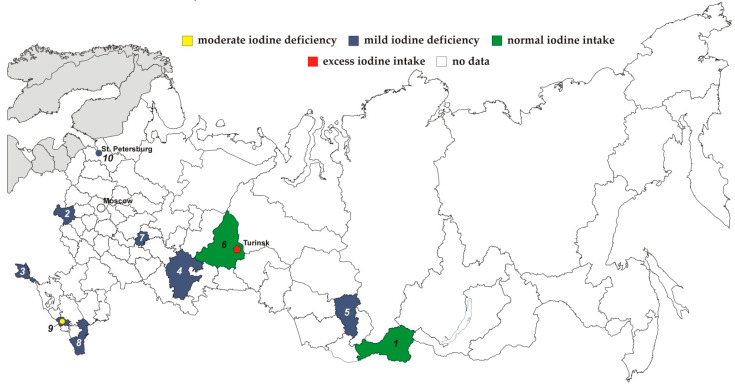
Iodine status among 6–12-year-old children in Russia. 1—Republic of Tyva; 2—Bryansk region; 3—Republic of Crimea; 4—Republic of Bashkortostan; 5—Kemerovo region; 6—Sverdlovsk region; 7—Republic of Chuvash; 8—Republic of Dagestan; 9—Republic of Kabardino-Balkaria; 10—St. Petersburg (city).

Median UIC ranged from 46 μg/L in the mountainous areas Republic of Kabardino-Balkaria [20] to 719 μ/L in Turinsk, Sverdlovsk region [14]. No geographical pattern in iodine status, if it exists, was observed due to the lack of data from most regions (Figure 2).

The World Health Organization (WHO) recommends screening of ID among 6–12-year-old children with the following classification based on the mUIC values [5]:

- <20 μg/L—severe ID;
- 20–49 μg/L—moderate ID;
- 50–99 μg/L—mild ID;
- 100–300 μg/L—normal iodine intake;
- 200–299 μg/L—more than adequate;
- >300 μg/L—excess iodine intake.

Normal iodine status was reported from 2 federal subjects, namely, Sverdlovsk region [13] and Republic of Tyva [16]. Mild ID was recorded in Bryansk [17] and Kemerovo [19] regions, Republics of Bashkortostan [10], Crimea [18], Chuvashia [20], Dagestan [8], Kabardino-Balkaria [14] and the city of St. Petersburg [15]. Moderate ID was reported only in mountainous areas of Kabardino-Balkaria [14]. Excess of iodine (719 μg/L) was registered in the town of Turinsk (Sverdlovsk region) [13].

## 4. Discussion

This is the first to our knowledge attempt to systematically summarize the evidence on the iodine status of among 6–12-year-old children in Russia. 

Iodine deficiency can occur at any age, but schoolchildren are the most commonly used population group for monitoring purposes. The WHO recommends 6–12-year-old children for monitoring purposes as a proxy for iodine status in the population [5]. Most of the international studies as well as the most recent Russian studies include 8–10-year-old children only [16,17,18]. 

Iodine deficiency in children is associated with difficulties at school and socialization problems [21]. School-based studies provide a valuable source of information on population iodine status keeping in mind susceptibility of school children in ID and relatively simple recruiting procedures. Although in settings with considerable proportion of children not attending schools these studies may overlook the iodine status among the most vulnerable children, this is not the case for Russia where more than 99% of children attend schools. School-based studies are recommended for monitoring purposes in regions where severe or mild ID was previously reported [22].

Unlike the mild iodine deficiency among adults and children in Russia stated in the IGN report our review provides the evidence on variations between the regions where the information on ID was available. Median UIC ranged from 46 μg/L in the mountainous areas Republic of Kabardino-Balkaria [14] to 719 μg/L in the town of Turinsk in the Urals. Moreover, high levels of UIC in Turinsk were registered during several years [13]. Tap water was considered to be the source of excess iodine in Turinsk, but little has been done to improve the situation according to the available literature [8]. 

Several studies from Asia and Africa have reported variations in the prevalence of goiter and mUIC in both children and adults by altitude [23]. ID has been shown to be more prevalent in high altitude settings. Soils in mountains are generally poor in iodine, thus food crops on these soils are poor in iodine [24]. Moreover, settlements located in the mountains are difficult to reach resulting in low accessibility of fortified foods [25]. Our result from Kabardino-Balkaria is in line with the international evidence on variations in UIC and ID by altitude. mUIC of 46 μg/L have been observed in mountainous areas of Kabardino-Balkaria, respectively, while the corresponding values for lowlands was 79 μg/L [14].

Despite the existing recommendations on monitoring of iodine status in regions with ID, the revealed in 2003 severe ID in Volgograd (17 μg/L) was not followed up in literature [21]. At the same time there is evidence on the effectiveness of ID prevention programs in some remote areas. The prevention program in the Republic of Dagestan included information campaign on the importance of iodine and recommended intake in mass media, use of iodized salt in mass caterers and free distribution of iodine supplements at antenatal care centres and schools [8]. However, the effectiveness of campaigns was limited reporting an increase in median UIC from 31–36 μg/L in 2002 to 66–75 μg/L in 2012 in coastal areas and from 23–30 μg/L to 65–69 μg/L in non-coastal towns over the same period reflecting a shift from moderate to mild ID [8].

The Republic of Bashkortostan assessed iodine status in children in 2013, 2015 and 2018 [10,11,12]. In all three studies rural-urban variations in iodine status were detected. Moreover, in 2 studies, the effect of ID prevention was reported. Schoolchildren in both urban and rural areas received free fortified milk twice a week from 2011 to 2014. In rural areas, using iodized milk for 9 months has led to an increase in mUIC from 52 μg/L to 121 μg/L among 8–10 years old children. The corresponding increase in urban areas was from 88 μg/L to 159 μg/L. At the same time the description of the campaign and how it how its results were evaluated was limited. Nevertheless, these two examples demonstrate the potential of the programs to prevent ID by introduction of one fortified product into the diet.

The absence of the national regulatory act on prevention of ID and no centralized monitoring system can explain missing information on UIC/ID in most federal subjects of Russia and substantial variations in both UIC and preventive activities [26]. Although iodized salt has been shown to be an effective measure for ID in many parts of the world, Russia introduced obligatory use of iodized salt in school catering only in 2020 [27,28]. Sporadic local and regional efforts to fortify bread, dairy products and other food items have also been reported, but there is no evidence on the effectiveness of these measures in Russian settings except one case in Bashkortostan. The National Medical Endocrinology Research Center promoted implementation of regional programs of ID prevention since 2005, but no scientific evaluation of their effectiveness is available. Additionally, an action plan was developed to implement the Strategy for improving the quality of food products in the Russian Federation until 2030 [27]. The Federal Law "On the prevention of diseases caused by iodine deficiency" was developed in 2018 but has not been implemented yet. The effectiveness of these steps remains to be assessed.

Methodological aspects are important for detecting ID and producing comparable data. Inductively coupled plasma mass spectrometry (ICP-MS) provides the most valid results of urine iodine concentration. Although this method is resource consuming [29], it is used in the Arctic biomonitoring program [30]. Most of the results summarized in this review were obtained using ceric-arsenite reaction as recommended by the WHO (Sandell–Kolthoff method) [31]. It has been shown that UIC obtained by Sandell–Kolthoff method correlates well with the gold standard [29]. However, a few studies used other methods or have not reported any method at all [10,12,19]. 

Other difficulties we encountered in analysis of existing evidence on iodine status of 6–12-year-old children in Russia included poor description of study design, no justification of sample size in most studies and differences in reporting of the results in terms of measures of central tendency and measures of variability as well as stratification by gender, age etc. 

More research with standardized methodology is needed to assess the prevalence of ID and its determinants in all federal subjects of Russia using the WHO recommendations to ensure comparability of the findings. Regional and even local data are crucial for tailoring public health measures to prevent or combat ID. Moreover, a scientific evaluation of all measures directed at prevention of ID is warranted. 

## 5. Conclusions

Our review demonstrates that there is no up-to-date data on iodine status of 6–12 years old children in almost 90% of federal subjects of the Russian Federation. The wide variation in the median UIC from moderate to excessive in the identified studies warrants research in other regions using standardized methodology. Most of the regions with available information suggest that Russia is a country with mild ID, but regions with moderate ID and excessive iodine intake were also identified. Systematic assessment and monitoring of ID in all regions across the country is warranted to obtain high quality data for development and evaluation of evidence-based national and region-specific public health programs to improve the situation.

## Figures and Tables

**Table 1:** Summary of research papers presenting urinary iodine concentration (UIC) among 6–12-year-old children in Russia published in 2013–2021.

Area of Residence (Number on the Map)	Year	n	Age, years	Indicator			UIC Assessment Methods	Reference
				mUIC (μg/L)	Interquartile Range (Q1;Q3)	Note		
Republic of Tyva (1)	2021	227	8–10	153			cerium-arsenite reaction	[16]
Bryansk region (2)	2021	337	8–10	98			cerium-arsenite reaction	[17]
Republic of Crimea (3)	2020	356	8–10	97			cerium-arsenite reaction	[18]
Republic of Bashkortostan (4)	2018	180	8–9	89 52	(45;144) (26;79)	town village	photometry (set of reagents «Merck», Germany)	[10]
Kemerovo region (5)	2016		7–10	98			potentiometry using ion-selective electrodes	[19]
Republic of Bashkortostan (4)	2015	181	8–10	88 159 61 121	(23;156) (60;191) (22;71) (98;162)	town after IS ^a^ village after IS	cerium-arsenite reaction	[11]
Sverdlovsk region (6)	2015	97 100	8–12 8–11	121 ^b^ 719		overall Turinsk	cerium-arsenite reaction	[13]
Republic of Chuvash (7)	2015		7–12	72			cerium-arsenite reaction	[20]
Republic of Dagestan (8)	2014		8–10	66–75 65–69		coastal towns non-coastal towns	cerium-arsenite reaction	[8]
Republic of Bashkortostan (4)	2013	181	8–10	89 159 52 121		town: before IS after IS village: before IS after IS	colorimetry (set of reagents «Merck», Germany)	[12]
Republic of Kabardino-Balkaria (9)	2013	1782	7–11	79 ^c^ 71 ^d^ 46 ^e^	(29;119) (39;132) (12;108)		cerium-arsenite by Saundell-Kolthoff reaction	[14]
St. Petersburg (10)	2013	883	6–10	68 65 61		overall boys girls	cerium-arsenite reaction	[15]

^a^ IS—iodine supplementation; ^b^—the author of the study [13] singled out a separate city Turinsk and when calculating the mUIC for the region as a whole, did not take into account the value obtained in Turinsk; ^c^—plain; ^d^—foothill; ^e^—mountain.
